# A Bayesian optimization approach for rapidly mapping residual network function in stroke

**DOI:** 10.1093/brain/awab109

**Published:** 2021-03-16

**Authors:** Romy Lorenz, Michelle Johal, Frederic Dick, Adam Hampshire, Robert Leech, Fatemeh Geranmayeh

**Affiliations:** 1 MRC Cognition and Brain Sciences Unit, University of Cambridge, Cambridge CB2 7EF, UK; 2 Stanford University, Stanford, CA 94305, USA; 3 Max-Planck Institute for Human Cognitive and Brain Sciences, Leipzig 04303, Germany; 4 Computational, Cognitive and Clinical Neuroimaging Laboratory, Department of Brain Sciences, Imperial College London, London W12 0NN, UK; 5 Birkbeck/UCL Centre for Neuroimaging, Birkbeck University, London WC1H 0AP, UK; 6 Centre for Neuroimaging Science, King’s College London, London SE5 8AF, UK

**Keywords:** chronic stroke, cognition, functional neuroimaging, closed-loop, machine learning

## Abstract

Post-stroke cognitive and linguistic impairments are debilitating conditions, with limited therapeutic options. Domain-general brain networks play an important role in stroke recovery and characterizing their residual function with functional MRI has the potential to yield biomarkers capable of guiding patient-specific rehabilitation. However, this is challenging as such detailed characterization requires testing patients on multitudes of cognitive tasks in the scanner, rendering experimental sessions unfeasibly lengthy. Thus, the current status quo in clinical neuroimaging research involves testing patients on a very limited number of tasks, in the hope that it will reveal a useful neuroimaging biomarker for the whole cohort. Given the great heterogeneity among stroke patients and the volume of possible tasks this approach is unsustainable. Advancing task-based functional MRI biomarker discovery requires a paradigm shift in order to be able to swiftly characterize residual network activity in individual patients using a diverse range of cognitive tasks. Here, we overcome this problem by leveraging neuroadaptive Bayesian optimization, an approach combining real-time functional MRI with machine-learning, by intelligently searching across many tasks, this approach rapidly maps out patient-specific profiles of residual domain-general network function. We used this technique in a cross-sectional study with 11 left-hemispheric stroke patients with chronic aphasia (four female, age ± standard deviation: 59 ± 10.9 years) and 14 healthy, age-matched control subjects (eight female, age ± standard deviation: 55.6 ± 6.8 years). To assess intra-subject reliability of the functional profiles obtained, we conducted two independent runs per subject, for which the algorithm was entirely reinitialized. Our results demonstrate that this technique is both feasible and robust, yielding reliable patient-specific functional profiles. Moreover, we show that group-level results are not representative of patient-specific results. Whereas controls have highly similar profiles, patients show idiosyncratic profiles of network abnormalities that are associated with behavioural performance. In summary, our study highlights the importance of moving beyond traditional ‘one-size-fits-all’ approaches where patients are treated as one group and single tasks are used. Our approach can be extended to diverse brain networks and combined with brain stimulation or other therapeutics, thereby opening new avenues for precision medicine targeting a diverse range of neurological and psychiatric conditions.

## Teaser

Stroke survivors require therapies that are tailored to their unique brain injury, but it is currently difficult to assess the degree to which damaged brain networks are engaged in mental activities. Lorenz et al. reveal how a novel brain-scanning tool can overcome this challenge in patients, paving the way to individualised therapy.

## Introduction

Cognitive and linguistic impairments following brain injury such as stroke are a leading cause of disability, affecting over a quarter of a million people in the UK, with numbers expected to increase dramatically given the ageing population.^1^ Current therapeutic strategies are only of limited success^2-4^; therefore, there is a need for developing biomarkers that guide clinical prognosis as well as rehabilitation strategies. Given the great heterogeneity in stroke patients, functional MRI is a promising method for discovering candidate biomarkers capable of distinguishing patient subgroups as it allows non-invasive mapping of brain (dys)function. However, to date, no functional MRI-derived biomarker is ready to be used in clinical trials for predicting recovery of cognitive or language function.^5^

Nonetheless, functional MRI measures during task execution (‘task-based functional MRI’) show promising potential as clinically relevant biomarkers and thereby represent a developmental priority.^5^ A major challenge for any progress in this direction is selecting the optimal task (or battery of tasks) to be administered to patients in the magnetic resonance scanner.

This is because neither cognitive nor language-related functions can be readily mapped to distinct, single brain regions but rather emerge through the interaction between domain-specific (e.g. motor, auditory, language networks) and domain-general brain systems. Highly domain-general brain networks, such as frontoparietal networks (FPNs) support processes including attention, working memory and learning (or re-acquisition) of a skill.^6–9^ Damage to domain-general brain networks may explain why cognitive impairments seen in stroke are distributed across diverse cognitive processes.^10^^,^^11^ We have previously shown that intact domain-general brain regions are critical in recovery of language function following aphasic stroke^12–15^ in keeping with studies confirming their role in recovery of motor deficits^16^ and the learning of pseudo language.^17^ This builds a convincing case for their potential as a prognostic biomarker.

However, characterizing residual function of domain-general networks in stroke patients is challenging because there is not a single, optimal task that is unique to probe each network; instead it involves quantifying network activation across many different cognitive tasks. However, such prolonged, multi-task neuroimaging protocols^18–20^ are practically unfeasible in patients. Thus, the current status quo for clinical neuroimaging studies typically involves selecting a specific task (or small subset of tasks) in a relatively *ad hoc* manner, in the hope that it will reveal a useful neuroimaging biomarker. Given both the sheer volume of possible tasks and the constraints on patient time, this approach is unsuitable. Advancing task-based functional MRI biomarker discovery requires a paradigm shift in order to be able to swiftly characterize residual brain network activity in individual patients using a diverse range of cognitive tasks.

**Figure 1:**
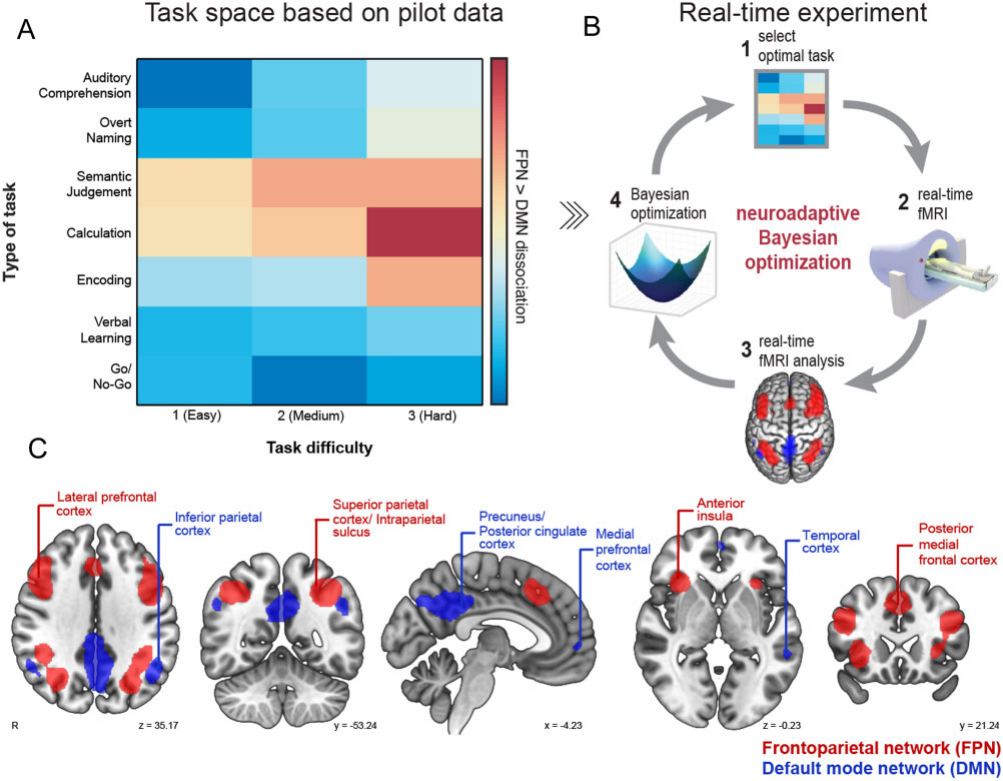
**Overview of methodology.** (**A**) We designed a 2D task space with one dimension corresponding to type of task (seven tasks), and the other to task difficulty (three levels). Tasks were ordered using pilot data collected separately in healthy volunteers. (**B**) This task space was searched through in our real-time experiment using neuroadaptive Bayesian optimization. The aim was to quickly identify a subject-specific set of tasks that maximize the difference in activity between the FPN and DMN. The method operates in the following steps: (1) the algorithm chooses a specific task × difficulty combination from the task space; (2) functional MRI data are collected while the subject is performing the task; (3) the difference in brain level activation between FPN (red) and DMN (blue) is computed in real-time; and (4) the result from step 3 is used to update the algorithm and subsequently choose the next task × difficulty combination to be presented to the subject in the next iteration [back to step (1) in a closed-loop fashion]. (**C**) FPN and DMN network masks derived from Yeo *et al.*^24^

Development of real-time analysis of functional MRI data in combination with machine-learning techniques (i.e. Bayesian optimization)^21^^,^^22^ provides an unprecedented opportunity to derive subject-specific profiles of brain network function across multiple tasks in a short period of time,^23^ making it feasible to use in patients. Neuroadaptive Bayesian optimization can efficiently search a large task space (Fig. 1A) to identify the optimal set of cognitive tasks that maximize a predefined target brain network state in each individual (Fig. 1B). The approach’s efficiency stems from the intelligent search procedure: based on real-time analysis of the functional MRI data, the machine-learning algorithm decides which task to test next in that particular subject; this is substantially faster than exhaustively testing all possible tasks while far more informative than selecting tasks at random.

Here, we apply neuroadaptive Bayesian optimization to a cohort of left-hemispheric stroke patients with chronic aphasia and demonstrate the approach’s potential for assessing patient-specific residual brain network function effectively and rapidly. Specifically, for each patient we identify the set of cognitive tasks that maximally dissociate two domain-general networks, namely increasing activation in the bilateral FPN, and decreasing activation in the default mode network (DMN) (Fig. 1C). The choice of this target brain state (i.e. FPN > DMN) was motivated by evidence suggesting that the difference in activity between these two networks was associated with language performance in left-hemispheric, aphasic stroke patients.^15^ For comparison, the method was also run in age-matched, healthy control subjects.

## Materials and methods

### Participants

The study was approved by the National Research Ethics Service Committee. We recruited 14 patients with left hemisphere infarcts, over the age of 40 [mean age ± standard deviation (SD): 58.57 ± 10.43 years, mean post-stroke time ± SD: 5.52 ± 3.25 years] and premorbid fluency in English. Patients with a previous history of a stroke resulting in aphasia or other neurological illness, or concurrent use of psychoactive drugs, were not eligible to enter the study. Table 1 contains further patient details. As controls, we recruited 15 fluent English-speaking healthy participants over the age of 40 (mean age ± SD: 56.73 ± 6.76 years), with no history of any neurological/psychiatric disorders. Sample size was informed by our previous studies using this technique in healthy individuals.^21^^,^^25^ All participants were right-handed, had normal or corrected-to-normal vision and normal adult hearing. For three patients (Patients 030, 032 and 040) the second run was discarded due to distress and/or fatigue, causing us to stop the run prematurely. These three patients were excluded from all analyses as we could not guarantee the validity of the patient-specific results. In addition, from the 15 control subjects, one had to be excluded as auditory stimuli could not be heard due to a technical issue. Thus, all analyses are based on 11 patients (four female, mean age ± SD: 59 ± 10.9 years, mean post-stroke time ± SD: 5.95 ± 3.42 years) and 14 controls (eight female, mean age ± SD: 55.6 ± 6.8 years).

**Table 1:** Details of stroke patients

Patient ID	Age	Sex	Time since stroke, in years	Lesion territory	Lesion volume, in cm^3^	CAT score Average/sum
030^a^	69	Female	6	SC, WM, C (FC, IC, PC, TC)	22.28	119.0/10.82
031	58	Male	11.5	C (left PC, TC), SC, WM	12.63	511.0/46.45
032^a^	50	Male	1.5	C (left FC, TC, PC), SC regions	17.81	307.0/27.91
033	63	Male	5.5	C (left FC, PC), WM	4.87	595.0/54.09
034	40	Male	0.5	Left SC WM	0.31	805.0/73.18
035	60	Female	5.5	SC WM, SC GM, C (FC, TC, PC, OFC)	21.64	424.5/38.59
036	78	Female	6.5	SC WM, C (FC, TC, PC, OFC)	1.96	573.5/52.14
037	52	Female	4.5	C (left FC, IC, PC), WM	14.34	558.0/50.73
038	55	Male	5	C (FC, IC, PC), WM, SC GM	4.58	702.5/63.86
039	72	Male	2.5	C (left FC, IC, PC), WM, SC GM	8.55	627.5/57.05
040^a^	52	Male	4.25	C (left FC, IC, WM), right-sided pontine WM	3.33	612.5/55.68
041	67	Female	4.5	C (left PC)	1.52	590.5/53.68
042	47	Male	12	C (left FC, IC and PC)	21.42	422.5/38.41
043	57	Male	7.5	C (left FC, IC, PC), WM, SC GM	12.76	179.0/16.27

C = cortical; FC = frontal cortex; GM = grey matter; IC = insular cortex; OFC = orbitofrontal cortex; PC = parietal cortex; SC = subcortical; TC = temporal cortex; WM = white matter.

a Patients were excluded from all analyses as their second run had to be prematurely stopped.

### Task space

Neuroadaptive Bayesian optimization is substantially more efficient than randomly or exhaustively sampling all tasks because of two desirable properties: (i) it incorporates prior information about how the cognitive tasks relate to each other; and (ii) guides its own sampling trajectory across tasks in an intelligent manner. The intuition behind (i) is that tasks that are expected to elicit a similar brain response are grouped together in the search space, while dissimilar tasks are grouped further apart. Thanks to this prior information, the algorithm does not need to test all possible tasks in the real-time optimization run, but instead can sample a few, highly informative tasks and then make predictions for all other tasks by applying a non-linear spatial regression (i.e. Gaussian process regression). This allows the algorithm to swiftly identify regions in the task space that are suboptimal for its optimization aim (i.e. maximizing FPN > DMN dissociation) and instead focus on sampling tasks from the optimal regions in the search space.

Here, we designed a 2D task space (Fig. 1A) with one dimension corresponding to ‘type of task’ and the other to ‘task difficulty’. We selected three cognitive tasks (Calculation, Go/No-Go, and Encoding) and four language tasks (Overt Naming, Auditory Comprehension, Semantic Judgement, and Verbal Learning). Tasks were chosen according to three criteria: (i) their ability to assess core cognitive and language deficits; (ii) their predicted probability of recruiting the FPN^24^; and (iii) the ability for patients to perform and understand these tasks.^26^ Whereas in our past work, we have aligned tasks in the search space based on a previous meta-analysis,^23^ here we added three tasks (Auditory Comprehension, Semantic Judgement and Verbal Learning) that were not part of this meta-analysis. Therefore, to order these seven tasks along the task dimension, we used pilot data collected prior to the real-time study in eight healthy volunteers (three female, mean age ± SD: 27.9 ± 8.3 years). Each task had three levels of difficulty with increasing complexity and cognitive demand, resulting in a total of 21 different task × difficulty conditions the algorithm could choose from. All tasks and their variants are briefly described in the Supplementarymaterial and depicted in Supplementary Fig. 1.

### Experimental procedure

All patients underwent the Comprehensive Aphasia Test (CAT) outside of the scanner^27^ before the experiment began (Supplementarymaterial).

For the real-time functional MRI study, each participant underwent two, independent optimization runs for which the algorithm was reinitialized and thus blind to any data collected in the subject’s previous run or any previous subjects, allowing us to assess the intra-subject reliability of results.

Each run was initiated randomly (i.e. first four task blocks were selected randomly from across the task space). The start of each run was synced with the onset of the first repetition time and each new task block was initiated by a repetition time. The first task commenced after 10 repetition times to allow for T_1_ equilibration effects. Each run lasted 14.2 min and consisted of 16 task block iterations; each iteration consisted of a task block lasting 34 s followed by 10 s rest block (white fixation cross on black background). Preceding each task block, participants received a brief instruction (5 s) about the task they would need to perform in the upcoming block followed by a short 3 s rest period (black background). For five patients, task instructions had to be provided orally via a microphone because of reading impairments. Participants used their left hand to indicate answers via a keypad.

Subjects were trained on all tasks outside of the scanner and informed about the real-time nature of the functional MRI experiment, but no information was given on the actual aim of the study or which parameters would be adapted in real-time. The investigator was not blinded due to the complexity of data acquisition and the need to ensure that real-time optimization was functioning.

### Real-time functional MRI

Masks of the bilateral target brain networks (Fig. 1C) were based on a meta-analysis reported in Yeo *et al*.^24^ The FPN (i.e. component 09) covered the superior parietal cortex, intraparietal sulcus, lateral prefrontal cortex, anterior insula and the posterior medial frontal cortex. The DMN (i.e. component 10) spanned the posterior cingulate cortex, precuneus, inferior parietal cortex, temporal cortex and medial prefrontal cortex. Thresholded (*z* > 2) and binarized maps of the two brain networks were used as mask.

Real-time functional MRI data analyses were performed on a conventional Mac mini system; in the Supplementary material we detail hardware specifications as well as the exact procedure for turning on the real-time export of functional MRI data on the Siemens magnetic resonance console computer. For real-time functional MRI preprocessing (Supplementarymaterial), we followed a similar procedure as described in our previous work.^23^ For computing the FPN > DMN dissociation target measure, after each task block, we ran incremental general linear models (GLMs) (Supplementarymaterial) on the preprocessed time courses of each network separately and then computed the difference between the estimates of all task regressors of interest (i.e. beta coefficients) for the FPN and DMN (i.e. FPN > DMN). The resulting contrast values were then entered into the Bayesian optimization algorithm. An initial burn-in phase of four randomly selected tasks was employed, i.e. the first GLM was only computed at the end of the fourth block after which the closed-loop experiment commenced.

### Bayesian optimization

Bayesian optimization is a two-stage procedure that repeats iteratively in a closed loop. The first stage is the data modelling stage, in which the algorithm uses all available samples obtained from real-time functional MRI (i.e. FPN > DMN contrast values) up to that iteration to predict the subject’s brain response across the entire task space using Gaussian process regression.^28–30^ For the Gaussian process, we used a zero mean function and the squared exponential kernel.^29^ The second stage is the guided search stage, in which an acquisition function is used to propose the task the subject will need to perform in the next iteration. Here we used the upper-confidence bound (GP-UCB) acquisition function^31^ that favours the selection of points with high predicted mean value (i.e. optimal tasks), but equally prefers points with high variance (i.e. tasks worth exploring). Algorithmic details for both stages are provided in the Supplementarymaterial.

### Statistical analysis

#### Behavioural accuracy

To assess if patients understood task instructions and performed higher than chance-level on the various tasks, we computed the non-parametric effect size measure AUROC (area under the receiver operating characteristic curve^32^) between the true empirical distribution of patients’ accuracy and the generated chance-level distribution for each task condition separately. The empirical distribution was computed for each task condition separately based on the mean accuracy (i.e. not AUROC) of each patient. The chance-level distribution was derived by randomly shuffling (1000 permutations) the trial sequence and the corresponding behavioural responses of each task block, and then re-computing the mean accuracy of each patient; this procedure had the advantage of preserving the overall response pattern of each patient. AUROC is one of the few existing non-parametric measures of effect size; thereby robust to violations of normality and advised for small samples. AUROC can be understood as a measure of overlap between two distributions and its values range from 0 to 1; a value at 0.5 indicates that there is no effect found between the two distributions (i.e. chance-level performance). Significance was determined when the one-sided lower 95% confidence bound (computed via bootstrapping) was higher than an AUROC of 0.5.

#### Linear mixed-effect models of behavioural and functional MRI data

**Figure 2:**
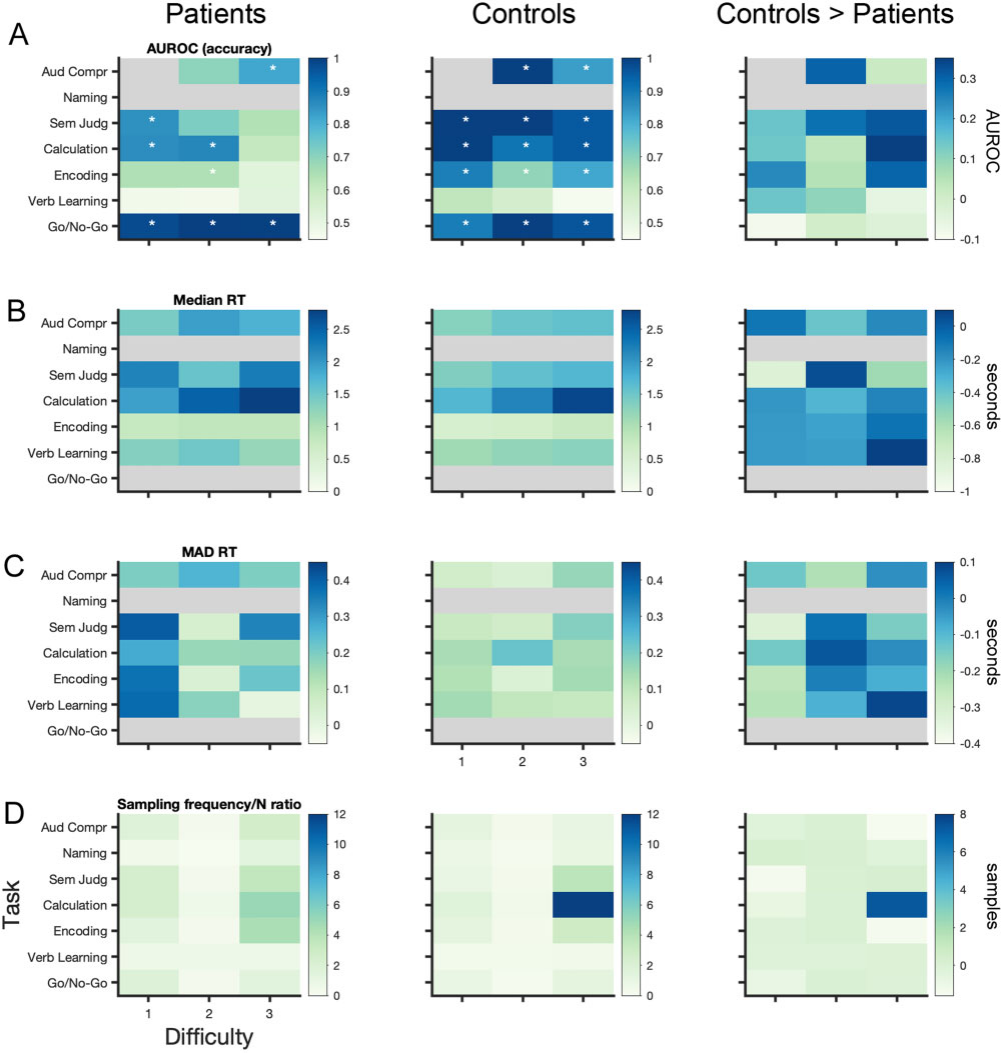
**Behavioural results and sampling behaviour of algorithm.** All results are listed for patients (*left*), controls (*middle*) and the difference between controls and patients (*right*). (**A**) AUROC is a non-parametric effect size measure indicating the difference between the empirically obtained accuracy and chance level for each task. Stars indicate the tasks for which patients and controls performed significantly above chance; significance was determined using a one-sided 95% lower confidence bound criterium of AUROC > 0.5, exact values are listed in Supplementary Table 1. (**B**) Median and (**C**) variance of reaction time [i.e. median absolute deviation (MAD)] across task space. (**D**) Given the algorithm’s subject-specific trajectories through the task space, each subject is exposed to a different set of tasks; here, we show the absolute number of times each task was selected by the algorithm—corrected for the difference in sample size *n* across both groups (i.e. *n* = 11 for patients and *n* = 14 for control subjects). Grey shaded areas correspond to NaN: accuracy could not be computed for the Naming task (as no chance level could be determined) and the first difficulty level of the Auditory Comprehension (as it was always correct); reaction time could not be computed in the Naming task (as no button press was required) and for Go/No-Go tasks (as subjects were instructed to inhibit a response). See Supplementary material for details on each task.

To assess behavioural performance, functional MRI measures, in-scanner motion and the relationship between functional MRI and behaviour, linear mixed-effect (LME) modelling was performed. As difficulty level 2 was sampled far fewer times than difficulty levels 1 and 3 (Fig. 2D), results from difficulty levels 1 and 2 were merged for these analyses. Several LME models were specified (by modelling interactions among fixed effects and adding/dropping random effects) for each dependent variable and model selection (details in the Supplementarymaterial) was performed using simulated likelihood ratio tests (with 500 replications for simulation and alpha level set at 0.05); each winning model as well as the number of competitor models tested against are listed in Table 2. For non-significant group-level LME results (i.e. no difference found between patients and controls), we performed equivalence testing using the two one-sided tests procedure^33^ with an alpha set to 0.05 to confirm the absence of a group-level effect. For this, the smallest effect size of interest was determined for each research question based on objective criteria and/or heuristics, which are detailed in the Supplementarymaterial.

**Table 2:** LME models for each dependent variable

Domain	Dependent variable	‘Winner’ LME model (based on simulated likelihood ratio tests)	Won competitions/# competitor models
Behaviour	(A1) **Accuracy** (% of correct trials within a task block)	Acc ∼ **1 **+** Group × Run** + **Group × Difficulty** + (*1 | Subject*) + (*1 | Task*) + (*1 | Subject: Task*)	7/7
	(A2) **Reaction times** (correct responses)	Rt ∼ **1 **+** Group × Run** + **Group × Difficulty** + (*1 | Subject*) + (*1 | Task*) + (*1 | Subject: Task*)	3/3
	(A3) **MAD accuracy**	MAD_Acc ∼ 1 + **Group × Run** + **Group × Difficulty** + **Run × Difficulty** + **Group × Run × Difficulty** + (*1 | Task*) *+* (*1|Subject*)	4/4
	(A4) **MAD reaction times**	MAD_Rt ∼ **1 **+** Group × Difficulty** *+* (*1|Subject*)	7/7
Functional MRI	(B1) **FPN > DMN**	betas_FPN>DMN ∼ **1 **+** Run** + **Group × Task** + **Group × Difficulty** + **Task × Difficulty** + **Group: Task: Difficulty** + (*1 | Subject*) + (*1 | Subject: Task*) + (*1 | Repetition*)	11/11
	(B2) **FPN**	betas_FPN ∼ **1 **+** Run** + **Group × Task** + **Group × Difficulty** + **Task × Difficulty** + **Group: Task: Difficulty** + (*1 | Subject*)	5/5
	(B3) **DMN**	betas_DMN ∼ 1 + **Group × Run** + **Group × Difficulty** + (*1 | Subject*) + (*1 | Task*) + (*1 | Subject: Task*)	4/5
	(B4) **MAD FPN > DMN**	MAD_betas_FPN>DMN ∼ **1 **+** Group × Run +** (*1 | Subject*)	7/7
	(B5) **In-scanner motion** (mean framewise displacement FD)	FD ∼ **1 **+** Group** + **Run** + (*1 | Subject*)	2/2
Functional MRI and behaviour	(C1) **Accuracy and FPN>DMN**	Acc ∼ **1 **+** Run** + **Difficulty** + **Group × betas_FPN>DMN** + (*1 | Subject*) + (*1 | Task*) + (*1 | Repetition*) + (*1 | Subject: Task*)	18/19
	(C2) **Reaction time and FPN > DMN**	Rt ∼ **1 **+** Run** + **Difficulty** + **Group × betas_FPN > DMN** + (*1 | Subject*) + (*1 | Task*) + (*1 | Subject: Task*)	13

Acc = accuracy; FD = framewise displacement; Rt = reaction time. For LMEs, categorical regressors were ‘Group’ (patients or controls), ‘Subject’ (our 25 different subjects), ‘Run’ (run 1 or run 2) and ‘Task’ (the seven tasks). Ordinal regressors were ‘Difficulty’ (difficulty level 1 or 3), and ‘Repetition’ (corresponding to the number of times the same task has been sampled before in an individual subject’s run). For the LME formulas in the third column of the table, fixed effects are indicated in bold and random effects are in italics.

#### Intra-subject reliability

To assess intra-subject reliability of our results, we computed the Spearman’s rank correlation of the Bayesian predictions across the task space between the two runs of each subject. For statistical inference, we performed permutation testing (i.e. 10 000 permutations) where we shuffled the FPN > DMN values and corresponding task indices of the second run and then refitted the Gaussian process (hyperparameters were kept identical to the real-time scenario) for these shuffled values before computing the correlation coefficient between the two runs. For each permutation, we then computed the median of the Fisher *z*-transformed correlation values for each group separately. To correct for multiple comparisons, at each permutation we only kept the maximum of both median values (i.e. ‘max statistic’ method^34^). The median of our true empirically obtained (Fisher *z*-transformed) correlation coefficients for patients and controls were then compared to the generated null distribution of maximum median values with a one-sided alpha-level set at 0.05.

#### Assessing dissimilarity of patients’ functional profiles

To assess if patients’ individual profiles were more dissimilar among each other than those of control subjects, we computed a dissimilarity matrix, i.e. the correlation distance (1 − Spearman’s rank correlation^35^) of each subject’s functional profile to all other subjects’ individual profiles. As intra-subject reliability was high across runs (see the ‘Results’ section), we derived each individual’s functional profile by collapsing both runs (i.e. fitting Gaussian process on all observations from both runs) with the aim of deriving a more precise depiction of individuals’ functional profiles. Next, we computed the correlation distance among all subjects. For statistical inference, we performed permutation testing (10 000 permutations) where we replicated this procedure but randomly shuffled the label for patients and controls. We then computed two different *t*-statistics: (i) the difference between controls’ dissimilarity (i.e. upper triangle of control-by-control matrix) and patients’ dissimilarity (i.e. upper triangle of patient-by-patient matrix); and (ii) the difference between controls-by-patient dissimilarity (i.e. full matrix) and patients’ dissimilarity. Finally, our true empirical *t*-statistics were then compared to the generated null distribution of *t*-values with a one-sided alpha-level set at 0.05. To visualize dissimilarity among patients’ functional profiles in 2D, we used classical multidimensional scaling (MDS), a dimensionality reduction technique that preserves between-subject distances. The technique can be understood analogous to a principal component analysis on the dissimilarity matrix, yielding the main principal coordinates through the data (i.e. eigenvectors); with the first one explaining most variance (i.e. largest eigenvalue). For subject clustering, we performed density-based spatial clustering of applications with noise (DBSCAN) on the dissimilarity matrix, an approach that groups together points that are closely packed together (i.e. points with many neighbours) and marks outliers that lie alone in low-density regions (i.e. with nearest neighbours too far). Cluster results were visualized in 2D MDS space. For comparison to MDS, we also computed the *t*-distributed stochastic neighbour embedding (t-SNE) on the dissimilarity matrix, a non-linear dimensionality reduction technique that better preserves the global structure of the data at the cost of between-subject distances.

To explore whether the dissimilarity among subjects’ functional profiles may be associated with performance, we took subjects’ weightings on the first principal coordinate of MDS and correlated (i.e. Pearson *r*) it with in-scanner and out-of-scanner behaviour. Since each subject had performed different tasks and difficulty levels in the scanner, mean in-scanner accuracy could not be obtained by simple averaging across all tasks; thus, we obtained mean in-scanner accuracy by extracting each subject’s random intercept from an LME (LME A1 in Table 2 but excluding the ‘Group’ regressor). For out-of-scanner performance, we used the sum of patients’ CAT score. To account for patients’ lesion volume (Table 1), we also ran partial correlation analyses. For statistical inference of these correlation analyses, we performed permutation testing (50 000 permutations, one-sided alpha-level set at 0.05) by randomly shuffling subjects’ labels; *P*-values obtained from this were corrected for multiple comparisons using the false-discovery-rate (FDR)^36^ with an alpha-level set at 0.05.

#### Normalizing patient’s FPN > DMN contrast and accuracy to control distribution

To assess if there was a single task, for which all patients showed a different FPN > DMN dissociation than controls, we ‘normalized’ the Bayesian prediction of each patient’s FPN > DMN contrast value to the control distribution using the modified *z*-score.^37^ This analysis was done for each task and difficulty level separately. This procedure was also performed for patient’s accuracy. However, in contrast to FPN > DMN contrast values for which we had Bayesian predictions for each task (i.e. Gaussian process regression across the task space), we did not have each subject’s accuracy for each task condition due to the sampling behaviour of the algorithm (Fig. 2D). Therefore, we limited the normalization of patient’s accuracy to task conditions for which we had enough controls (i.e. *n* > 7) to accurately compute the control distribution. A patient’s FPN > DMN contrast value and accuracy for a particular task was marked significantly different (i.e. ‘outlier’) when the absolute modified *z*-score was >1.96. This was a liberal criterion, as commonly a threshold of 3.5 is used.

### Data availability

All Python, bash and MATLAB code for implementing neuroadaptive Bayesian optimization is available from GitHub http://github.com/romylorenz/strokeLoop. For Gaussian process regression, we use a Python implementation from http://github.com/SheffieldML/GPy. Relevant data are available from the authors upon reasonable request.

## Results

### Most patients are able to perform multiple tasks in the scanner

Given the nature of the clinical populations, we first assessed whether patients performed above chance while undergoing the scan, indicating their understanding of the various task instructions. For this, non-parametric effect size measures (i.e. AUROC) were computed for each task condition separately, comparing patient group-level accuracy with chance level. Results (Fig. 2A, left, corresponding lower confidence bound is listed in Supplementary Table 1) demonstrate that patients performed above chance for all difficulty levels of the Go/No-Go task, for the easiest and medium levels of the Calculation task and the easiest level of the Semantic Judgement task. Further, they performed above chance for the most difficult level of the Auditory Comprehension task but not for the medium level—which can likely be explained due to unequal sampling across both conditions (i.e. the medium level was only sampled three times) (Fig. 2D). With respect to the Encoding task, patients performed only above chance for the medium difficulty level. Given that the lower confidence bound for the easiest level of the Encoding task is 0.4903 (Supplementary Table 1) and AUROC values are >0.6 for the easiest and medium level of this task, it can be assumed that patients performed higher than chance—even though it appears that this task is among the harder tasks tested. Patients did not perform above chance for any level of the Verbal Learning task, however, neither did controls (Fig. 2A, middle), illustrating that this task was ill-designed (Supplementarymaterial).

### Patients perform less accurately, slower and more variably than control subjects

As expected, overall patients performed less accurately [LME A1 (all LME formulas listed in Table 2): Group *t*(746) = −4.13, *P* < 0.001] and slower than controls [LME A2: Group *t*(643) = 5.53, *P* < 0.001]. Median and variance [i.e. median absolute deviation (MAD)] of reaction times for each task are shown in Fig. 2B and C, respectively. Both patients and controls performed less accurately [LME A1: Difficulty *t*(746) = −4.76, *P* < 0.001] and more slowly for more difficult task levels [LME A2: Difficulty *t*(643) = 9.84, *P* < 0.001]. Patients’ accuracy was not differentially affected by task difficulty compared to controls [i.e. no interaction effect, LME A1: Group × Difficulty *t*(746) = −0.95, *P* < 0.034]; in fact, they showed a gentler increment in response time with increasing difficulty compared to controls [LME A2: Group × Difficulty *t*(643) = −2.58, *P* = 0.01]. This is due to patients’ considerably slower responses for the easiest task conditions relative to controls (Fig. 2B) and that there was a set time window to respond for each task (Supplementarymaterial). Whereas reaction times decreased from the first to second run in both groups [LME A2: Run *t*(643) = −2.98, *P* = 0.003], only patients were more accurate in the second run [LME A1: Group × Run *t*(746) = 2.04, *P* = 0.042]. Overall, we found that patients showed a trend to vary more in their within-task accuracy than controls [LME A3: Group *t*(100) = 1.97, *P* = 0.052]. Accuracy in both groups varied more in the second versus first run [LME A3: Run *t*(100) = 2.33, *P* = 0.022], but this effect seems to be driven by an increase in variance for controls rather than patients [LME A3: Group × Run *t*(100) = −3.05, *P* = 0.003], but only for the easiest task conditions [LME A3: Group × Run × Difficulty *t*(100) = 2.42, *P* = 0.017] as across both groups, variability of accuracy decreased in the second run for the most difficult task conditions [LME A3: Run × Difficulty *t*(100) = −2.47, *P* = 0.015]. We found no effects of within-task variance in reaction times (LME A4) between both groups; however, we could not confirm that the group-level effect was statistically equivalent [*t*(16.33) = 0.48, *P* = 0.319], given symmetric equivalence bounds of ± 0.6 in standardized Cohen’s *d* effect size.

### Neuroadaptive Bayesian optimization is a feasible technique for patients

With respect to our real-time optimization results of FPN > DMN dissociation across the task space, we found significant intra-subject reliability for controls (median Spearman rho ± SD: 0.91 ± 0.18, *P* < 0.001) and patients (0.71 ± 0.45, *P* < 0.001). When investigating how FPN > DMN contrast values varied for the same task within an individual (i.e. when sampled multiple times), we found no significant difference in variance between patients and controls [LME B4: Group *t*(108) = 0.88, *P* = 0.38], this effect was statistically equivalent [*t*(14.71) = 1.99, *P* = 0.033], given symmetric equivalence bounds of ±1.2 in Cohen’s *d* effect size. With respect to in-scanner motion, we found that both patients and controls moved significantly more in the second run [LME B5: Run *t*(47) = 2.34, *P* = 0.023] but that there was no significant difference between the two groups [LME B5: Group *t*(47) = 1.74, *P* = 0.088]. We confirmed this effect to be statistically equivalent [*t*(14.42) = 2.425, *P* = 0.015] given symmetric equivalence bounds of ±0.2 mm on a raw scale. This indicates robustness of our obtained results and demonstrates the feasibility of the approach to achieve reliable results in patient populations.

### Semantic judgement, calculation and encoding tasks maximally dissociate FPN from DMN in patients and controls

**Figure 3:**
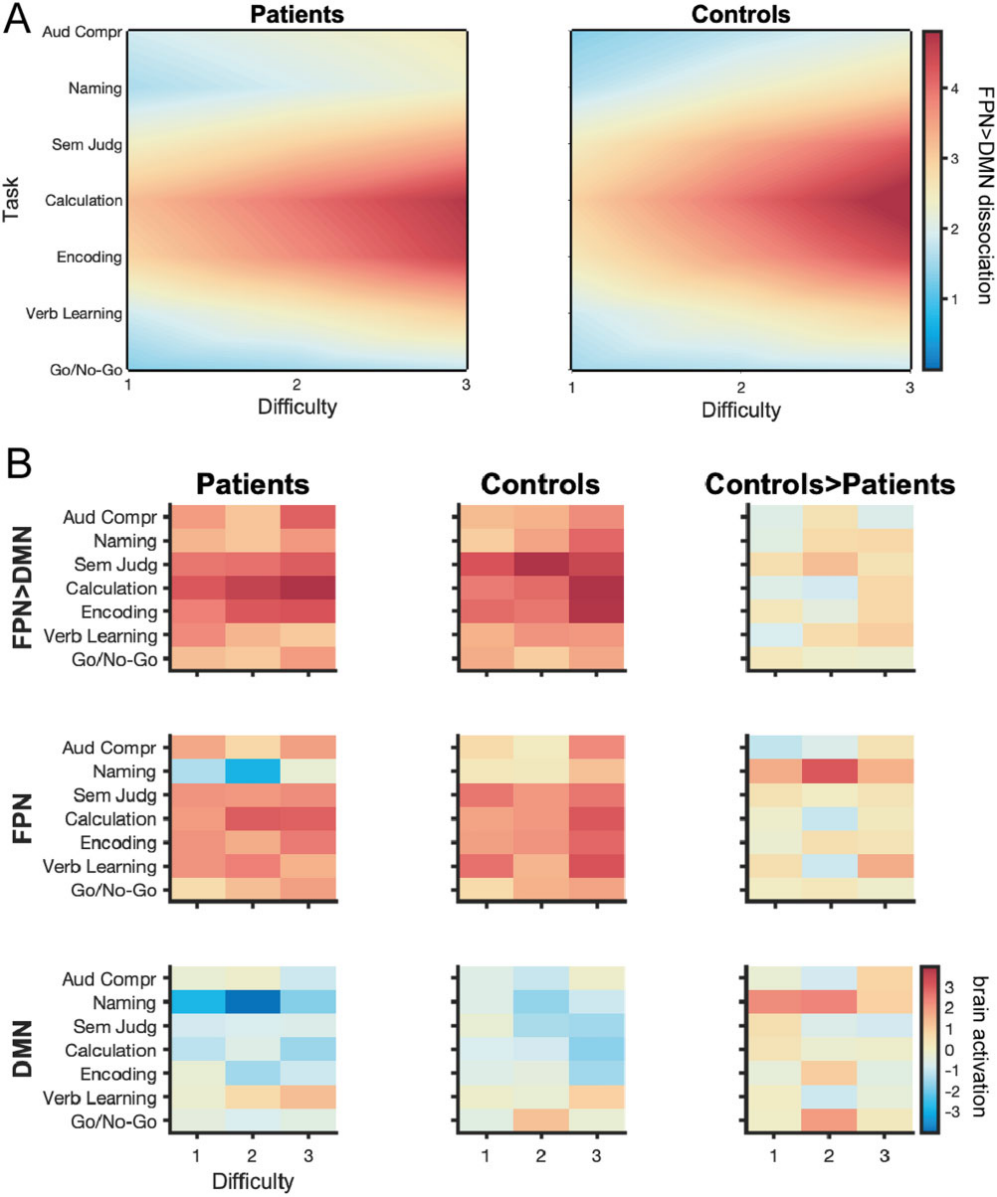
**Group-level results of real-time optimization.** (**A**) Group-level Bayesian predictions across task space (i.e. Gaussian process regression across all observations) for patients (*left*) and controls (*right*) indicate no qualitative difference in the FPN > DMN dissociation pattern across the task space between both groups. Patients appear to only have a slightly diminished FPN > DMN dissociation for the Semantic Judgement, Calculation and Encoding tasks. (**B**) To confirm that Bayesian predictions in **A** are not driven by the specific hyperparameters of the Gaussian process regression (Supplementary material), we also plotted the median of the FPN > DMN dissociation values across the task space for both groups (*top row*). We confirm that the Bayesian predictions appropriately capture the underlying distribution of median FPN > DMN contrast values. To understand the relative contribution of the FPN and DMN to our group-level results, we plot the brain activation values for those networks separately (*second and third rows*).

Group-level Bayesian predictions across the task space (i.e. Gaussian process regression on all observations) are shown in Fig. 3A for patients and controls, separately. We found that across both groups, Semantic Judgement [LME B1: *t*(771) = 5.11, *P* < 0.001], Calculation [LME B1: *t*(771) = 4.39, *P* < 0.001] and Encoding [LME B1: *t*(771) = 4.26, *P* < 0.001] tasks maximally differentiate the FPN from the DMN. Collapsed over all tasks, more difficult task conditions result in a larger FPN-DMN dissociation in both groups [LME B1: Difficulty *t*(771) = 2.95, *P* = 0.003]. The sampling behaviour of the Bayesian optimization algorithm clearly confirms these results (Fig. 2D): for both patients and controls the most difficult conditions of these three tasks were most often selected by the algorithm, indicating that the algorithm identified them as optimal for maximizing the FPN > DMN dissociation. While this is very pronounced for controls, in particular for difficulty level 3 of the Calculation task (Fig. 2D, middle); it is worth noting that the algorithms sampled much more exhaustively across the task space for patients (Fig. 2D, left), potentially indicating more diversity in the optima identified among individual patients. Surprisingly, at the group level, it appears that patients do not show a qualitatively different FPN-DMN dissociation pattern across the task space compared to controls (Fig. 3A), but only seem to have a slightly diminished FPN > DMN dissociation for the Semantic Judgement, Calculation and Encoding tasks. These qualitative observations are also confirmed statistically: patients have a significantly lower FPN > DMN dissociation only for the Semantic Judgement task independent of difficulty level [LME B1: Group × Semantic Judgement *t*(771) = −2.11, *P* = 0.035]. This finding may be because the patients’ group-level results are not a good representation of individual results of patients and is in line with the algorithm’s sampling behaviour. To understand the relative contribution of both the FPN and DMN to these results we also computed the activation values for both networks across the task space separately (Fig. 3B, second and third row). While we found no significant difference among the groups for either the FPN [LME B2: Group *t*(771) = 1.28, *P* = 0.20] or DMN [LME B3: Group *t*(794) = 0.61, *P* = 0.54], equivalence testing could not confirm the group-level effect to be statistically equivalent for either network [*t*_FPN_(10.56) = 0.76, *P* = 0.23, *t*_DMN_(10.4) = 0.35, *P* = 0.36, given symmetric equivalence bounds of ±0.5 in Cohen’s *d* effect size]. Finally, we were interested in understanding the relationship between neural and behavioural measures. While higher FPN > DMN contrast values were associated with longer reaction times across both groups [LME C2: FPN > DMN *t*(643) = 2.47, *P* = 0.014], there was no significant difference of this effect in patients [LME C2: Group × FPN > DMN *t*(643) = −1.31, *P* = 0.19]. Further, we did not find any association between the magnitude of FPN > DMN dissociation and accuracy across [LME C1: FPN > DMN *t*(746) = 1.59, *P* = 0.11] or between the two groups [LME C1: Group × FPN > DMN *t*(746) = 0.35, *P* = 0.73].

### Patients show unique profiles of network dysfunction

**Figure 4:**
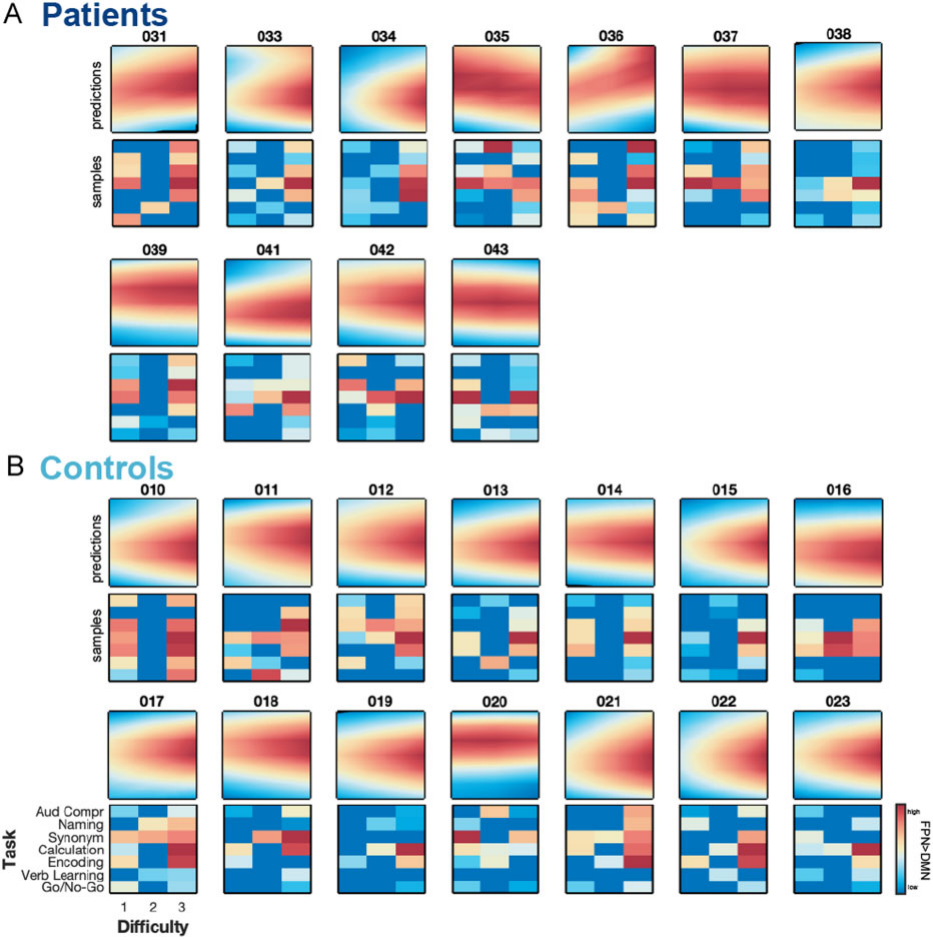
**Subject-level results of real-time optimization.** (**A**) Patients show unique profiles of FPN > DMN dissociation across the task space. (**B**) It can be clearly seen that in contrast to patients, controls show a striking similarity of FPN > DMN dissociation across the task space. For all patients and controls, we show the Bayesian predictions of FPN > DMN dissociation (i.e. Gaussian process regression based on subject-specific samples) across the entire task space (*top row*) as well as all samples individually (*bottom row*); when a task was sampled multiple times within a subject, we computed the median across those samples. We can see that Bayesian predictions appropriately capture the underlying distribution of samples.

Motivated by these findings, we wanted to understand if indeed patients’ real-time optimization results are more diverse than control subjects’ results. When looking at the dissimilarity of FPN > DMN profiles between patients (Fig. 4A), we found that they are significantly more dissimilar (*t* = −5.02, *P* = 0.038) than the FPN > DMN profiles between controls (Fig. 4B). Interestingly, we found that patients’ individual profiles are even more dissimilar amongst each other than when comparing them with controls’ individual profiles (*t* = −2.77, *P* = 0.024). These statistically significant findings demonstrate that patients really have unique profiles of network dysfunction but that some patients look more similar to controls than to other patients.

**Figure 5:**
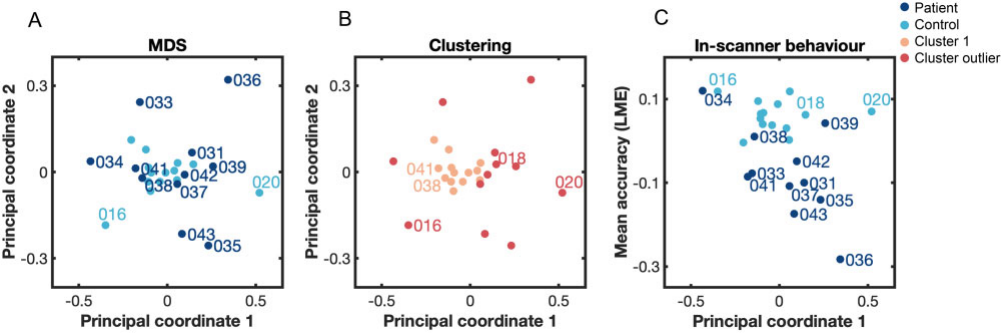
**Dissimilarity of functional profiles and association with behaviour.** (**A**) Visualization of dissimilarity (1 − Spearman) among each patient’s (dark blue) and control’s (turquoise) individual profile in 2D using MDS, a non-linear dimensionality reduction technique that preserves between-subject distances. Most controls (except Patients 016 and 020) cluster together at the centre, indicating high similarity between their functional profiles. In contrast, most patients lie dispersed around the cluster of healthy controls, indicating higher dissimilarity among their functional profiles. (**B**) Density-based clustering of dissimilarity (1 − Spearman) among each subject’s functional profile confirms descriptive results from **A**: we identified one dense cluster (peach) consisting of almost all controls (except Subjects 016, 018, 020) and two patients (Patients 038 and 041). All other nine patients were classified as ‘outliers’ of the cluster by the algorithm (red). (**C**) Subject’s variance on the first principal coordinate of MDS is significantly associated with their behavioural performance in the scanner (*r* = −0.40, *n *=* *25, *P* = 0.025/*P*_FDR_ = 0.042). As controls (turquoise) perform almost at ceiling, this association is mainly driven by patients (dark blue) and persists even when accounting for patients’ respective lesion volume (*r* = −0.63, *n* =* *11).

To visualize this finding, we plotted the dissimilarity among each patient’s and control’s individual profile in 2D using MDS, a dimensionality reduction technique that preserves between-subject distances. In Fig. 5A, we see that the majority of controls cluster together (turquoise) at the centre, indicating high similarity between their functional profiles. In contrast, most patients (dark blue) lie dispersed around the cluster of healthy controls and show higher variance along the first and second principal coordinates, indicating higher dissimilarity among their functional profiles. Using density-based clustering, we confirm these descriptive results: we identified one dense cluster (peach) consisting of most controls (11 of 14 controls, exceptions are: Subjects 016, 018 and 020) and two patients (Patients 038 and 041) while all other nine patients were classified as ‘outliers’ (red) of the cluster by the algorithm (Fig. 5B). We found that subjects within the cluster perform significantly better [*t*(23) = 2.17, *P* = 0.018] and less variable [two-sample *F*-test for equal variance, *F*(12,11) = 0.17, *P* = 0.002] on tasks in the scanner than subjects around the cluster; given that most controls fall into this cluster, these results are expected.

For comparison, we used another dimensionality reduction technique (t-SNE) that better preserves the global structure of the data at the cost of between-subject distances. We notice that t-SNE (Supplementary Fig. 2) pulls apart patients mainly based on their respective weighting on the first principal coordinate derived from MDS (Patients 033, 034, 038 and 041 have a negative weighting and are thus grouped closer to controls with t-SNE, while the other patients have a positive weighting). Thus, to explore whether variance on the MDS’s first principal coordinate (that explains most of the variance of dissimilarity between all subjects’ functional profiles) also relates to variance in behaviour, we simply correlate the weighting on the first coordinate with in-scanner behaviour (i.e. mean accuracy across all tasks performed in the scanner—derived by extracting each subject’s random intercept from an LME, see ‘Materials and methods’ section). We found a significant negative relationship (*r* = −0.40, *n *=* *25, *P* = 0.025/*P*_FDR_ = 0.042) across patients and controls; however, this seems to be mainly driven by patients given the controls’ performance approaching ceiling level (Fig. 5C). When only taking patients into account, this negative association is strengthened (*r* = −0.64, *n *=* *11, *P* = 0.016/*P*_FDR_ = 0.042) and remains high even when accounting for patients’ lesion volume (*r* = −0.63, *n *=* *11, *P* = 0.025/ *P*_FDR_ = 0.042). We found a moderate, yet not significant negative relationship with out-of-scanner behaviour (SupplementaryFig.3). Given our low sample size, we want to caution against the overinterpretation of these correlation results.

Since our patient cohort suffers from chronic post-stroke aphasia, we would expect that the result of patients exhibiting unique patterns of network function is not specific for the dissociation of the FPN from the DMN but also holds for functional networks classically associated with language. We tested this assumption and could replicate our results for a left-lateralized language network. By contrast, when focusing our analysis on a network associated with motor function, we found no significant difference between patients and controls (Supplementarymaterial). These supporting analyses illustrate our method’s specificity in characterizing individual level network dysfunction in patients.

#### The potential of single tasks for biomarker discovery is limited

**Figure 6:**
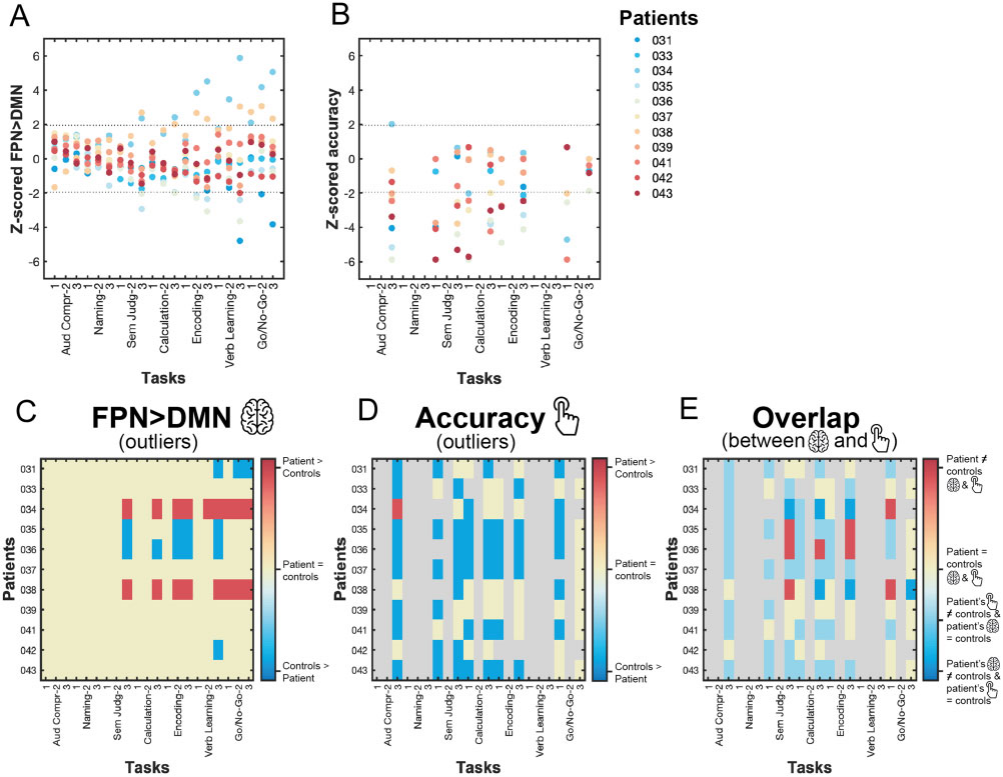
**Task-wise normalization (*z*-scoring) of patients’ FPN > DMN dissociation and accuracy to controls’ group result.** (**A**) Patients’ FPN > DMN Bayesian prediction values *z*-scored with respect to the control distribution for each task condition; each patient has a unique colour code. (**B**) Patients’ accuracy *z*-scored with respect to control distribution separately for each task condition; each patient has a unique colour code. Note that not all tasks could be z-scored because of a too small control sample for those tasks (refer to the ‘Materials and methods’ section). (**C**) A patient’s FPN > DMN contrast value for a particular task was marked significantly different (i.e. ‘outlier’, red or blue) when the absolute modified *z*-score was >1.96. For only 9 out of 21 possible task conditions, we identified at least a single patient that showed a dysfunctional FPN > DMN dissociation (i.e. weaker or stronger FPN > DMN dissociation than controls). Importantly, there are only a few tasks that show a dysfunctional FPN > DMN dissociation in the exact same subset of patients: (i) Patients 034, 035, 036 and 038 showed significantly different FPN > DMN contrast values for the medium and most difficult levels of the Encoding task and the most difficult level of the Semantic Judgement task; (ii) Patients 031, 034 and 038 showed a significantly different FPN > DMN dissociation for the medium and difficult level of the Go/No-Go task. Importantly, for more than half of the tasks (i.e. 12), we do not find a significantly different FPN > DMN dissociation for patients. (**D**) A patient’s accuracy was marked significantly different (i.e. ‘outlier’, red or blue) when the absolute modified *z*-score was >1.96. (**E**) When comparing the outlier results, we observed little correspondence between patient’s dysfunctional FPN > DMN dissociation and behaviour (red). Interestingly, patients that showed a significantly stronger FPN > DMN dissociation than controls (i.e. Subjects 034 and 038) seemed to perform similarly to controls on some of these tasks (dark blue). The majority of our patients, however, performed significantly worse than controls while showing no significant difference in FPN > DMN dissociation (light blue). Grey shaded areas correspond to NaN because of missing behavioural data for that subject (grey square) or because of a too small control sample for those tasks (grey column).

Functional profiles derived from real-time optimization seem suitable for inferring patient’s current behavioural capabilities, indicating their potential usefulness as clinically relevant biomarkers for predicting stroke recovery (i.e. a patient’s future functional capacity). To further understand if these multivariate profiles of residual network function yield additional information to that can be obtained from univariate analyses of individual task activations, as is conventionally performed in clinical neuroimaging research, we ‘normalized’ each patient’s task-specific Bayesian prediction values with respect to the controls’ distribution. Results from this analysis revealed the particular tasks on which each patient significantly deviates from healthy controls (Fig. 6A and C). We were able to identify difference in patients’ FPN > DMN dissociation from those of healthy controls, in only 9 of 21 possible task conditions. As those conditions include almost exclusively the hardest (five) and medium (three) difficulty levels, selecting an appropriate task difficulty level seems to play an important role in separating patients from controls. Moreover, different tasks identify different subsets of patients that display a significantly altered FPN > DMN dissociation compared to controls; this implies that task selection has an impact on which patients are labelled as deviating from controls. Importantly, when comparing these results to patients’ individual task performance (Fig. 6B and D), we found little resemblance (Fig. 6E): most patients performed significantly worse than controls on those tasks while showing no significant difference in their FPN > DMN dissociation (light blue). Taken together, these results highlight the challenge in *a priori* selecting an appropriate task and difficulty level, questioning the potential of univariate task-based functional MRI biomarkers for predicting stroke recovery.

## Discussion

In this study, we applied neuroadaptive Bayesian optimization for the first time to a cohort of patients with the aim of rapidly searching through a variety of different cognitive and language-related task conditions in order to obtain patient-specific functional profiles of residual domain-general network function.

At the group level, patients qualitatively did not show an altered FPN > DMN dissociation pattern across tasks compared to controls. For both patients and controls, more difficult task conditions and particularly the Semantic Judgement, Calculation and Encoding tasks maximally dissociated the FPN from the DMN. This is in line with our previous work, showing that the Calculation and Encoding tasks as well as increased task demands strongly recruit this particular FPN in healthy volunteers.^23^^,^^24^ While we found no significant difference between patients and controls for the Calculation and Encoding tasks, we did find the Semantic Judgement task to be associated with a significantly diminished FPN > DMN dissociation in patients on a group level. However, it should be noted that our sample size was primarily optimized for individual-level analyses (i.e. small sample with multiple runs per subject) and not for drawing group comparisons. Given that some of our equivalence tests yielded non-significant results, our study lacks power to detect smaller group-level differences. Additional analyses revealed though that even the existence of a group-level effect is not a prerequisite that a given tasks differentiates well between patients and controls on the individual level: when investigating individual patients’ neural responses for the Semantic Judgement task (i.e. the task that showed significantly different FPN > DMN dissociation between patients and controls on the group level), only two out of all patients showed a significantly lower FPN > DMN dissociation compared to controls and two other patients showed a significantly stronger FPN > DMN dissociation on that task compared to controls. This highlights the limitations involved with the conventional approach in clinical neuroimaging: it is remarkably difficult to predict before the start of a clinical study which task will reveal a sensitive biomarker that can be applied to an individual patient and that is capable of differentiating patients from controls as well as subgroups of patients; this is especially important considering the heterogeneity in patients with respect to lesion location and multiple co-morbidities (e.g. vascular disease) that differentially affect the function of brain networks.

At the subject-level, we confirmed the validity of patient-specific functional profiles by comparing the real-time optimization results of two independent runs. We found patient-specific profiles to be consistent; however, controls’ functional profiles were characterized by a much higher intra-subject reliability. This lower intra-subject reliability of patient-specific functional profiles may be explained by patients showing learning effects (improved accuracy) in the second run, potentially contributing to slightly different results across both runs.

Our findings clearly demonstrate that group-level results are not representative of individual patient-specific results. We found greater heterogeneity among the functional profiles of patients than among those of controls. In fact, patients feature idiosyncratic profiles of FPN > DMN dissociation across the task space. This was also in line with the sampling trajectories of the real-time algorithm that sampled much more diversely for patients. By contrast, the algorithm’s sampling was much more focused for controls as they showed a very high consistency in their functional profiles; a finding replicating our earlier work.^21^^,^^23^ Interestingly, we could show that the variance in patients’ functional profiles is associated with their in-scanner performance even when accounting for lesion volume. This indicates that patients’ functional profiles are indeed capturing a specific functional dysfunction that cannot be predicted from just the spatial extent of their stroke lesion. Given our low sample size, we are convinced that studies with larger sample size and study preregistration are needed to corroborate these explorative findings. Such studies will be able to shed light on whether patients’ functional profiles represent a (multidimensional) continuum or can be classified into subgroups (e.g. patients with positive versus negative loading on the first principal coordinate of MDS). Equally, such studies could investigate why a few healthy control subjects also show considerable variance in their functional profiles. While speculative, functional profiles may be suitable markers for detecting early cognitive decline that has not displayed in major behavioural deficits yet. While we have only looked at patient’s relative differences of FPN > DMN magnitude among tasks (i.e. 1 − Spearman correlation across tasks space) and compared them to controls, future studies could also take absolute FPN > DMN differences into account (e.g. by using 1 − Euclidean distance across task space^35^) as in the univariate analyses, we observed two particularly well-performing patients who showed a much stronger FPN > DMN dissociation compared to controls.

One challenge of this closed-loop experimental framework is that subjects are required to remember various task instructions and switch between tasks in a relatively swift manner (every minute in our case). Despite these heightened task demands, in our study, patients performed above chance for all tasks on at least the easiest difficulty level (an exception was the Verbal Learning task for which also control subjects performed at chance).

In summary, our study highlights the importance of moving beyond traditional ‘one-size-fits-all’ approaches in clinical neuroimaging where patients are treated as one group and single tasks (or a few tasks) are used. Instead, we demonstrate that mapping residual network activity following brain injury across many different tasks using real-time optimization yields robust patient-specific functional profiles that carry meaningful information about a patient’s behavioural capacity. From a conceptual point of view, this multi-task approach also improves the generalizability of our findings^38^ because the Gaussian process explicitly models the subjects’ brain response across many different cognitive tasks and variants of the same task (e.g. different difficulty levels); results obtained are, therefore, not specific to a single task,^39^ allowing for far more principled generalization of these results. Thus, multivariate functional profiles of residual brain function derived from neuroadaptive Bayesian optimization may have promising potential to become clinically relevant and generalizable biomarkers with satisfactory test-retest reliability, that could be leveraged to make patient-specific predictions about recovery and guide individualized treatment planning.

Understanding how patient-specific profiles of residual network function could be utilized for predicting stroke recovery and guide rehabilitation is, however, beyond the scope of the present study and needs to be addressed in longitudinal studies with larger sample size. This would allow the study of how changes in patients’ profiles at different stages of stroke recovery relate to gradual behavioural and cognitive improvements. While we have employed the technique to stroke patients in the chronic stage of recovery, it can be equally administered to patients in the subacute phase of stroke. Considering that we found no univariate linear relationship between FPN > DMN dissociation and task accuracy in this study, we argue that the relationship between patient’s profiles of residual network function and behaviour is most likely of multivariate and possibly also non-linear nature. For example, so far it is not clear if therapy should focus on training patients on tasks associated with high or low residual FPN > DMN dissociation or tasks for which residual FPN > DMN dissociation is most different to controls. To unravel the possibly complex relationship between profiles of residual network function and behaviour, it may be critical to test patients on a large battery of tasks outside of the scanner, which would allow obtaining patient-specific multivariate behavioural profiles (e.g. Butler *et al*.^40^and Halai *et al*.^41^) with adequate statistical power, that then can be related to multivariate profiles of residual network function using machine-learning techniques such as canonical correlation analyses.

Neuroadaptive Bayesian optimization has immediate therapeutic potential in that it permits identifying patient-specific sets of tasks for training specific brain networks/states; these tasks could be administered as part of cognitive behavioural therapy over prolonged periods of time. Importantly, our approach can also be combined with therapeutic interventions involving non-invasive brain stimulation.^39^^,^^42^ Using neuroadaptive Bayesian optimization, cognitive task conditions and non-invasive brain stimulation parameters could be searched through simultaneously with the aim of identifying optimal therapeutic protocols tailored to individual patients for behavioural therapy (i.e. optimal task) in conjunction with brain stimulation (i.e. optimal stimulation intensity).

Since structural brain imaging has been shown to predict stroke patients’ current linguistic and cognitive impairments^43^ as well as language outcome and recovery,^44^^,^^45^ an avenue for future research would be to incorporate lesion information derived from structural scans (∼5 min) with rapidly obtainable functional profiles (∼0–15 min) to further boost the accuracy of such predictions. To what extent resting state functional MRI (e.g. Bonkhoff *et al*.^46^) may carry additional predictive value, is an outstanding scientific question. Equally, instead of using the same brain network masks for all patients as we have done here, the technique could be further refined by *a priori* specifying subgroup-specific brain networks masks (e.g. derived from left-handed stroke patients) or even individualized network masks derived from anatomical landmarks, functional localizers (e.g. Fedorenko *et al*.^47^ and Mahowald and Fedorenko^48^) or resting state functional MRI (e.g. Braga and Buckner^49^and Gordon *et al*.^50^).

The strength of our real-time optimization approach lies in the rapid mapping out of functional profiles of residual network function across a large space of cognition without the need to exhaustively sample all possible tasks. This efficiency makes it a highly interesting tool for clinical populations; yet it may come at a cost of sensitivity. For example, we identified four stroke patients whose functional profiles are similar to those of healthy controls. Therefore, an interesting future direction may be to use neuroadaptive Bayesian optimization as a first stage for obtaining a comprehensive yet coarse depiction of residual brain function. Results obtained from this first stage could then be used to inform a second stage of dense sampling^51–53^; patients could then be tested repeatedly over a long period of time on a subset of tasks identified with real-time optimization, or real-time optimization repeated repeatedly.^23^ Such a two-stage procedure would yield very precise individual functional profiles across the most informative tasks.

Beyond optimizing for brain network activation/dissociation as we have done here, neuroadaptive Bayesian optimization is highly versatile, allowing us to target any clinically promising functional brain state that can be estimated in real-time, for example based on functional^42^^,^^54^ or effective connectivity,^55^ multivariate patterns of functional MRI activation,^56–58^ or EEG.^59^^,^^60^ Selecting appropriate target brain states is ideally based on prior exploratory studies for identifying candidate functional neuroimaging-based biomarkers that carry predictive power about recovery and treatment response.^5^ From a statistical point of view, the lower the contrast-to-noise (CNR) ratio of the target brain state, the more observations (e.g. longer runs) are needed to obtain satisfactory accuracy^21^; therefore, target brain states for clinical populations should be chosen that have moderate-to-high CNR.

In conclusion, we show for the first time that neuroadaptive Bayesian optimization is a feasible, reliable and highly efficient approach for identifying patient-specific functional profiles of network dysfunction. While the sample size is currently small, we show that these unique patient profiles are associated with behaviour, thereby demonstrating the potential of this approach for exploring and testing novel neuroimaging biomarkers for recovery after stroke. This technique has broad reaching clinical implications and can be extended to a wide range of neurological and psychiatric conditions. In particular, it will be of interest to those developing presurgical functional localization around lesions such as epileptogenic focus or tumours. Furthermore, this approach can be extended to optimize for any target brain network/state and optimize task conditions and non-invasive brain stimulation parameters conjointly, thereby opening new avenues for precision medicine for a wide range of neurological disorders.

## Supplementary Material

awab109_Supplementary_Data

## Abbreviations

AUROC: area under the receiver operating characteristic curve
DMN: default mode network
FPN: frontoparietal network
LME: linear mixed effect
MAD: median absolute deviation
MDS: multidimensional scaling
