# Supplementary material for: A Bayesian optimization approach for rapidly mapping residual network function in stroke
Source: Brain. 2021 Mar 16;144(7):2120–34. doi: 10.1093/brain/awab109 (PMC8370405; doi:10.1093/brain/awab109)
Supplement: awab109_Supplementary_Data [file awab109_supplementary_data.pdf]

Supplementary Material

A Bayesian optimisation approach for rapidly mapping residual network function in stroke

Romy Lorenz, Michelle Johal, Frederic Dick, Adam Hampshire, Robert Leech & Fatemeh Geranmayeh

Supplementary Figure 1	Stimulus presentation of each task
Supplementary Figure 2	2D visualization of dissimilarity among functional profiles using t-SNE
Supplementary Figure 3	Relationship with out-of-scanner behaviour

Supplementary Table 1	One-sided 95% lower confidence bound for AUROC

Supplementary Methods 1	Task descriptions
Supplementary Methods 2	Comprehensive Aphasia Test (CAT)
Supplementary Methods 3	Real-time fMRI hardware specifications
Supplementary Methods 4	Real-time fMRI pre-processing
Supplementary Methods 5	Real-time fMRI incremental GLMs
Supplementary Methods 6	Algorithmic details of Bayesian optimization
Supplementary Methods 7	Details of LME model selection
Supplementary Methods 8	Equivalence testing

Supplementary Results 1	Supporting analyses of language and motor network

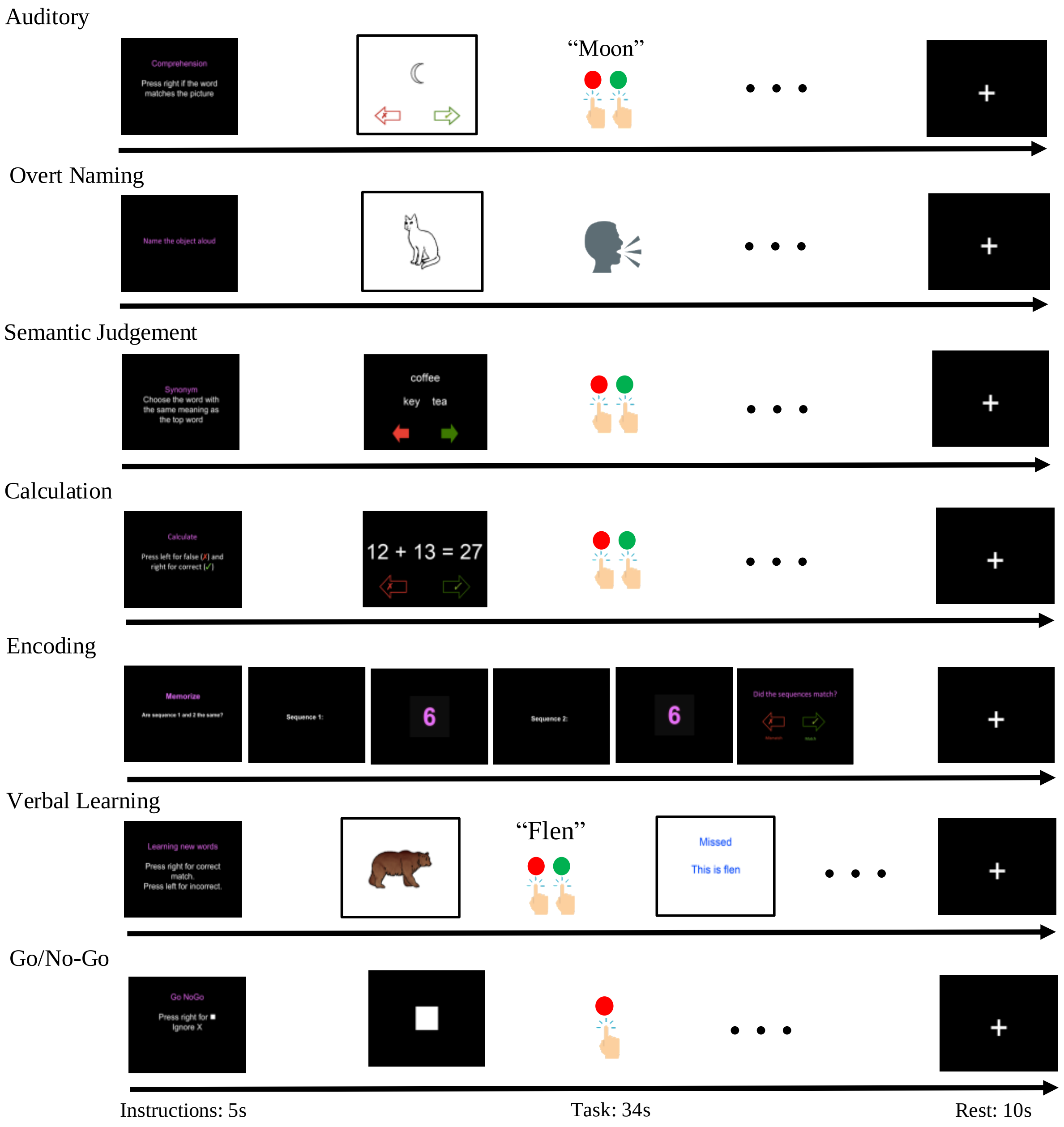

Supplementary Figure 1: **Stimulus presentation of each task**. Each task block lasted 34 s followed by 10 s rest. Preceding each task block, participants received a brief instruction (5 s) about the task they would need to perform in the upcoming block followed by a short 3 s rest period (black background, not shown here). Except the Overt Naming tasks, all tasks required participants indicating their responses using a keypad with their left hand. The left hand was chosen to avoid difficulties related to right sided motor impairments as is common in left middle cerebral artery stroke. The keypad had two buttons, the right button for correct answers and the left button for incorrect answers. Where only one button response was required the right was pressed.

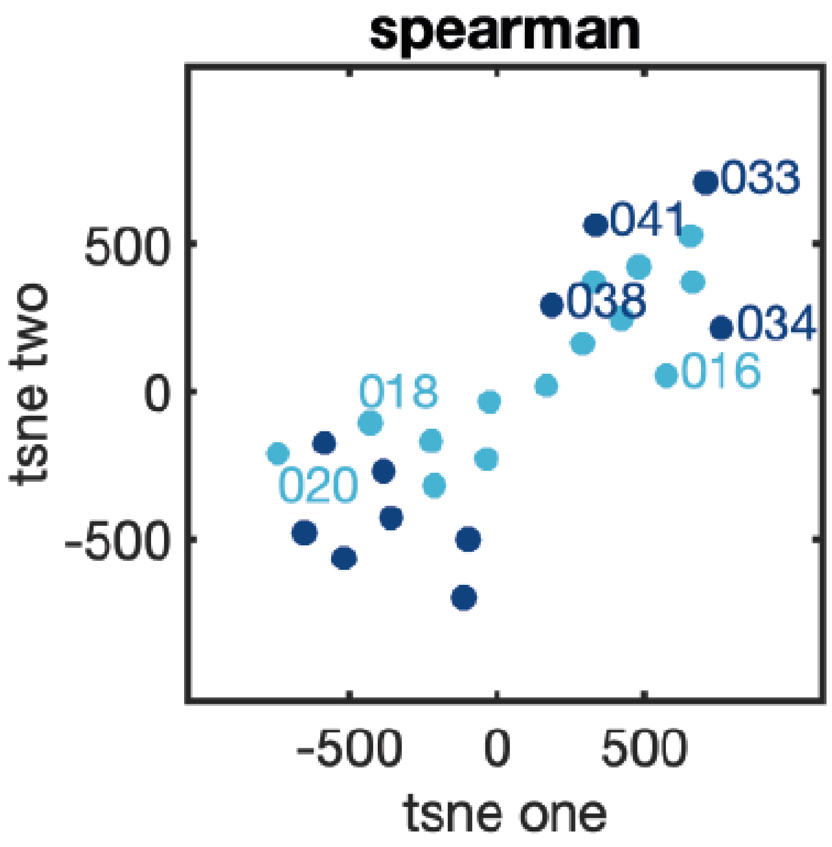

Supplementary Figure 2: 2D visualization of dissimilarity among functional profiles using t-SNE. In contrast to MDS, t-SNE better preserves the global structure of the data at the cost of between-subject distances. Using t-SNE we descriptively see that the majority of patients group together (dark blue) with the exception of four particular patients (033,034, 038 and 041) who are closer to controls' functional profiles (turquoise). In contrast, a single control subject (020) is grouped closer to patients than to other controls. Using different dimensionality reduction techniques (MDS and t-SNE), our results converge for two patients (038,041). Qualitatively, we observe a striking resemblance between those two patients' profiles (Fig. 4a in main text) and controls' profiles (Fig. 4b in main text) as they display the highest FPN>DMN dissociation on the most difficult level of the Calculation task. In contrast, between a single (020 with tSNE) and three (016, 018 and 20 with MDS) control subjects are grouped further away from other controls. Qualitatively, these controls seem to have in common, that unlike other controls, they show a high FPN>DMN dissociation for easier and medium task difficulty levels (Fig. 4b in main text). When comparing results from both techniques, we notice that tSNE pulls apart patients mainly based on their respective weighting on the first principal coordinate from MDS (patients 033,034, 038 and 041 have a negative weighting while the other patients have a positive weighting, Fig. 5a in main text).

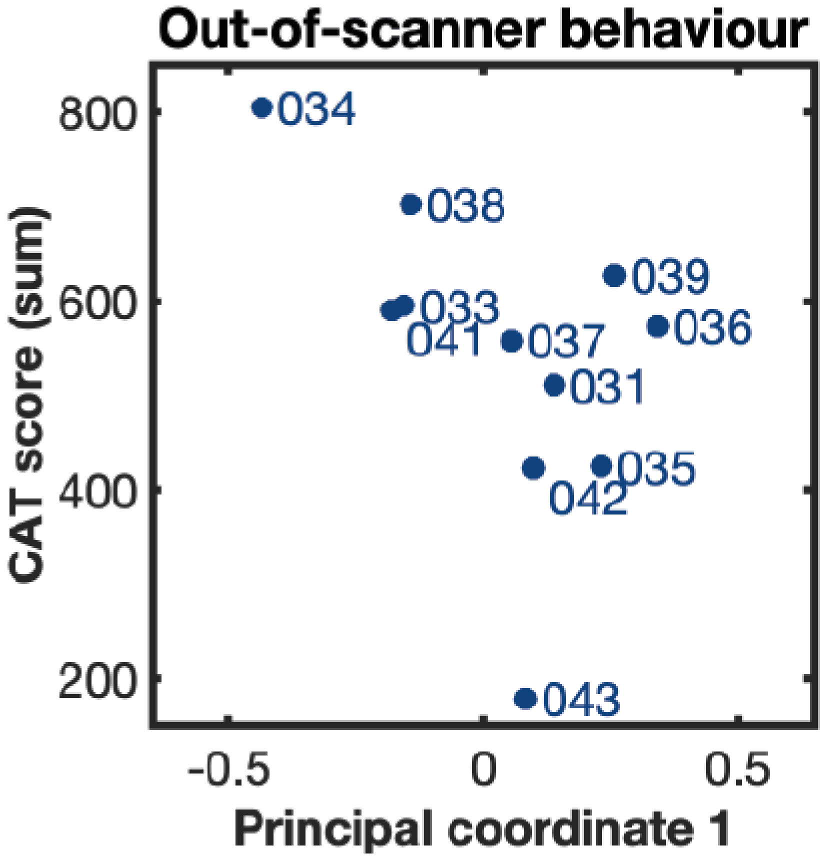

Supplementary Figure 3: Relationship with out-of-scanner behaviour. We found a moderate, yet not significant, negative correlation between the MDS' first principal coordinate and patient's CAT scores (r = -.51, n =11, p = .056/ p^{FDR} = .069); when accounting for lesion volume, this association is further weakened (r = -.28, n=11, p = .206 / p^{FDR}=.206).

Supplementary Table 1: One-sided 95% lower confidence bound for AUROC

	Patients			Controls		
**Task**	**Difficulty 1**	**Difficulty 2**	**Difficulty 3**	**Difficulty 1**	**Difficulty 2**	**Difficulty 3**
Auditory Comprehension	Nan	.4059	.7015	Nan	.9935	.664
Naming	Nan	Nan	Nan	Nan	Nan	Nan
Semantic Judgement	.6464	.4407	.4741	.9979	.9993	.8920
Calculation	.7250	.7884	.4011	.9946	.7802	.8813
Encoding	.4903	.5026	.3321	.7971	.5946	.7168
Verbal Learning	.2500	.3134	.4229	.4057	.3731	.3322
Go/No-Go	.9448	.9982	.9899	.6707	.9971	.9030

Supplementary Methods 1: Task descriptions

A 2D task space was designed consisting of seven different tasks with three difficulty levels each. All tasks and their variants are briefly described below and depicted in Supplementary Fig. 1.

Auditory Comprehension: This language task tested participants' ability to understand verbal word stimuli and match it to the correct picture. Correct pairings were indicated using a keypad. The word picture associations were based on the PALPA (Psycholinguistic Assessments of Language Processing in Aphasia). At difficulty level 1, 10 image-spoken word pairs were presented and pairings were always correct. At difficulty level 2, 14 image-spoken word pairs were presented and unrelated distractors were included. The probability of receiving an incorrect pairing was 50%. At difficulty level 3, close semantic distractors were included and the probability of receiving an incorrect pairing was 50%. Each image was shown for 3 s.

Overt Naming: This language task tested participants' ability to name objects. Participants were shown a series of images and required to verbalise (into a microphone) what the stimulus represented. Responses were listened to online to ensure participants performed the task as instructed, by attempting to verbalise at the correct times. Level 1 included easy words and level 2 included hard words. Stimuli were classified as easy or hard based on reaction times and name agreement data collected in a previous experiment (Krishnan *et al.*, 2015). At level 3, 25 words with the lowest frequency were used from the Graded Naming Test (McKenna and Warrington, 1983). As this task did not require any button responses and rather relied on the quality of the verbal output, chance level could not be determined.

Semantic Judgement: This language task tested participants' ability to decipher the meaning of words. It was designed by (Jefferies *et al.*, 2009) and included words scaled by their interpretation difficulty, based on imageability and frequency. Participants were required to indicate which two words (out of three) were semantically related using a keypad. Each trial appeared on screen for 3.5 s. At higher difficulty levels the words chosen had lower frequency and imageability.

Calculation: This non-language task tested participants' ability to perform mental arithmetic. Patients with aphasia often suffer from acalculia (Luccia and Ortiz, 2016), and this task is also known to rely heavily on the FPN (Yeo *et al.*, 2014; Lorenz *et al.*, 2018). In each equation, participants had to decide whether the answer provided was correct or incorrect using the keypad. At level 1, the equations involved one-digit addition and each equation was shown for 2.9 s, at level 2, it involved addition of numbers between 10–20. At level 3, it involved higher two-digit subtraction and each equation was shown for 3.5 s. The probability of receiving an incorrect answer was 50%.

Encoding: This non-language task tested participants' working memory through digit span. Each digit in a sequence appeared on a black background for 0.4 s with the next digit in the sequence appearing after a 0.1 s gap. This was then followed by a second sequence of numbers of the same length. Participants were required to indicate whether the second sequence matched the first using a keypad. At level 1, digit span was three, at level 2, digit span was 5 and at level 3, digit span was seven. The probability of receiving a mismatched number sequence was 50%.

Verbal Learning: This language task tested participants' ability to learn new words based on feedback. It was designed by (Sliwinska *et al.*, 2017) and was shown to rely heavily on the FPN. Participants were presented with a series of images with audio stimuli denoting a pseudo word (using English phonology) to each image. Without prior training, participants were required to indicate whether the image and word pairing was correct. They were then provided with feedback as to whether their response was correct and provided with the correct name. If

they did not respond, they still received feedback (Sliwinska *et al.*, 2017). Level 1 involved learning two correct pseudo word–object associations, level 2 involved learning four and level three involved learning 10. At each level 3, correct associations were mixed with 25 incorrect associations. However, with hindsight this task was not suited for the current study as for many pairings (in particular for level two and three), participants were not able to correctly learn the new pairings within the short blocks of 34 s. As a result, patients and healthy controls performed at chance on this task.

Go/No-Go: This non-language task tested participants' ability to identify targets and inhibit responses. In each trial either a white square, which required a "Go" response (pressing the right key on the keypad), or a cross which required a "No-Go" response (participants did nothing) were shown on screen. At level 1, stimuli were presented for 0.5 s with a 1 s gap, and 20% of trials were "Go". At level 2, stimuli were presented for 0.25 s with a 0.75 s gap, and 35% of trials were "Go". At level 3, a blue square was also incorporated, which participants were supposed to treat as a "No-Go" as well as the white cross. Stimuli were presented for 0.25 s with a 0.7 second gap, and 30% of trials were "Go".

Supplementary Methods 2: Comprehensive Aphasia Test (CAT)

The CAT provides measures for word fluency, comprehension of spoken language, comprehension of written language, repetition, naming, reading, writing, descriptive speaking, descriptive writing, as well as a cognitive score. All subscale scores were simply summed to provide a single measure of aphasic deficit for the patients.

Supplementary Methods 3: Real-time fMRI hardware specifications

Real-time computations were performed on a Mac Mini system (Late 2012) running on OS X Mavericks (10.9.5) with the following technical specifications: 2.3 GHz quad-Core Intel Core i7 processor, 16GB of 1600 MHz DDR3 SDRAM memory, 1TB Serial ATA hard drive storage and Intel HD Graphics 4000. The Mac Mini is connected to the MR scanner network through an ethernet cable.

At the console computer of the Siemens MR scanner, the Mac mini is mapped as a network drive, to which dicom files can be exported in real-time. The real-time export of dicom files can be turned on using the *ideacmdtool* on the MR console computer; mapping a network drive and using the ideacmdtool requires to switch into the *advanced user mode* at the MR console computer. In the ideacmdtool, the path to the Mac mini folder where the real-time dicom files are being expected (as specified by the real-time fMRI pre-processing scripts) can be specified under option *4 - Outline Export Defaults* (with "Port" set to -1). Finally, under option *5 - Switches*, "Send IMA" needs to be turned on. Note, that "Send IMA" commonly resets following the registration of a new patient on the MR console computer; however, this is the only step that needs to be turned on again after each new patient registration.

TR triggers from the scanner and presses from the button response box are received by the Mac Mini via USB interfaces. The Mac Mini system controls the presentation screen in the scanner via a thunderbolt to DVI adapter.

Supplementary Methods 4: Real-time fMRI pre-processing

Images with whole-brain coverage were acquired in real-time by a Siemens Verio 3 T scanner using an EPI sequence (T2*-weighted gradient echo, voxel size: 3.00 × 3.00 × 3.00 mm, field of view: 192 × 192 × 105 mm, flip angle: 80°, repetition time (TR)/echo time (TE): 2000/30 ms, 35 interleaved slices). Prior to the online run, a high-resolution gradient-echo T1-weighted

structural anatomical volume (voxel size: 1.00 × 1.00 × 1.00 mm, flip angle: 9°, TR/TE: 2300/2.98 ms, 160 ascending slices, inversion time: 900 ms) and one EPI volume were acquired. Online pre-processing was carried out with FSL (Jenkinson *et al.*, 2012). The first steps occurred offline prior to the real-time fMRI scan. Those comprised brain extraction using BET (Smith, 2002) of the structural image followed by a rigid-body registration of the functional to the downsampled structural image (2 mm) using boundary-based registration (Greve and Fischl, 2009) and subsequent affine registration to standard brain atlas (MNI) (Jenkinson and Smith, 2001; Jenkinson *et al.*, 2012). In patients with large lesions, BET was performed iteratively with various options (e.g., centre-of-gravity, fractional intensity threshold, vertical gradient in fractional intensity threshold) to allow the best brain extraction; results were inspected visually by experimenter. The resulting transformation matrix was used to register the FPN and DMN from MNI to the functional space of the respective subject. For online runs, incoming EPI images were motion corrected (Jenkinson *et al.*, 2002) in real-time with the previously obtained functional image acting as reference. In addition, images were spatially smoothed using a 5 mm FWHM Gaussian kernel. For each TR, means of the two brain networks were extracted. To remove outliers, scrubbing (i.e., data replacement by interpolation) was performed on these time courses with the cut-off set to ± 4 SD. Removal of low-frequency linear drift was achieved by adding a linear trend predictor to the general linear model (GLM). To further correct for motion, confound regressors were added to the GLM consisting of six head motion parameters and a binary regressor flagging motion spikes (defined as TRs for which the framewise displacement exceeded 3).

Supplementary Methods 5: Real-time fMRI incremental GLMs

Incremental GLM refers to the design matrix growing with each new task block, i.e., the number of timepoints as well as the number of regressors increasing with the progression of the real-time experiment. The GLM consisted of task regressors of interest (one regressor for each task block), task regressors of no interest (e.g., 5 s instruction period) as well as confound regressors (seven motion and linear trend regressor, see Supplementary Methods 3) and an intercept term. Task regressors were modelled by convolving a boxcar kernel with a canonical double-gamma hemodynamic response function (HRF). After each new block, the beta coefficients were re-estimated.

Supplementary Methods 6: Algorithmic details of Bayesian optimization

At the *data modelling* stage, our GP was defined by a zero mean function and the squared exponential kernel (Rasmussen and Williams, 2006) as covariance function:

$$[\mathbf{1}]\ \ k(x,y) = \sigma^2\, exp\, exp\left\{-\frac{(x-y)^2}{2\, l^2}\right\} + \sigma_{noise}^2$$

where $x, y \in R^2$ correspond to the choice of task condition. The hyperparameters $\sigma \in R$ and $l \in R^2$ each determine the variance and length scale of the covariance kernel, respectively. Further, it is assumed that observations are corrupted by white noise, σ_{noise}^2.

These hyperparameters had to be selected prior to running the real-time experiments; we used independent data from eight pilot subjects to tune these parameters using Type-2 maximum likelihood (Rasmussen and Williams, 2006). This choice of hyperparameters was then fixed for all participants' runs in the real-time study and the same hyperparameters were also used for all offline analyses reported here. The first four task blocks served as a burn-in for a first estimate of the GP.

At the *guided search* stage, we employed the upper-confidence bound (GP-UCB) acquisition function (Srinivas *et al.*, 2010):

$$[\mathbf{2}]\ UCB(x) = \mu(x) + \sqrt{v\tau_t}\sigma(x),$$

where

$$v = 1 \text{ and } \tau_t = 2log\,(\frac{t^{\frac{d}{2}+2}\pi^2}{3\delta}).$$

Formally, at every iteration, the next task is selected by maximising the GP-UCB:

$$[\mathbf{3}]\ x_{next} = argmax_x\{UCB(x)\}$$

This new sample will then be used to return to the first stage and update the GP; thereby closing the experiment loop.

Supplementary Methods 7: Details of LME model selection

We performed simulating likelihood ratio tests (as opposed to the likelihood ratio test) as it is recommended when testing for fixed effects and allows comparing arbitrary LME models, i.e., models do not need to be nested and can have different random and fixed effects. The final model was selected that "won" all or most simulated likelihood ratio tests against its competitor models. As conventionally done, winning was determined as $p < .05$ for the more complex model (using the *compare* function in Matlab 2019b). Only models were compared against each other that resulted in a positive definiteness of the Hessian of the objective function with respect to unconstrained parameters at convergence which was used as a criterion to verify optimality of the solution (this results in the different number of competitor models for each dependent variable). The LME formula of the winning models are listed in Table 2 in the main manuscript for each dependent variable separately alongside the number of competitor models tested and the number of won competitions.

Supplementary Methods 8: Equivalence testing

In case of non-significant group-level LME results (i.e., no effect found between stroke patients and controls), we tested for equivalence between the groups following the two one-sided tests (TOST) procedure. It is important to note that equivalence testing does not test whether there exists no effect at all between the groups, rather it examines whether the hypothesis that effects are extreme enough to be considered meaningful can be rejected (Lakens *et al.*, 2018). Performing TOST therefore involves determining the smallest effect size of interest (SESOI) for each test separately. The TOST procedure is then performed against lower and upper equivalence bounds that are specified based on the SESOI. Equivalence testing was performed in *RStudio* (Version 1.3.1093). In the following, we justify the equivalence bounds chosen for each equivalence test performed.

Given the non-significant group-level LME A.4 result, we tested for equivalence between the groups regarding within-task variance in reaction times (as measured by median absolute deviation (MAD)) by conducting TOST against equivalence bounds of $\Delta L = -0.6$ to $\Delta U = 0.6$ in standardized effect sizes (Cohen's d). The effect size of 0.6 was selected based on the literature: effect sizes of intra-individual variability in reaction time between elderly healthy controls (n=17) and Alzheimer patients (n=17) were shown to be large with d = 1.3 while it was moderate with d =.63 between elderly healthy controls (n=62) and patients suffering from mild cognitive impairment (n=55) (Phillips *et al.*, 2013). We chose the stricter equivalence

bound of d=0.6 as the majority of stroke patients from our cohort are not severely cognitively impaired.

Given the non-significant group-level LME B.4 result, we tested for equivalence between the groups regarding within-task variance in fMRI estimates (as measures by median absolute deviation) by conducting TOST against equivalence bounds of $\Delta L = -1.2$ to $\Delta U = 1.2$ in standardized effect sizes (Cohen's d). We chose a particular large effect size for this test as neuroadaptive Bayesian optimization is particularly well-suited for dealing with noisy observations (e.g., large within-task variability) due to two desirable properties: (1) the GP explicitly models noise and this parameter is tuned based on prior data (see equation [1] in Supplementary Methods 6) and (2) an acquisition function was chosen that favours points with high uncertainty around the predictive mean (see equation [2] in Supplementary Methods 6). This practically means that neuroadaptive Bayesian optimization chooses to sample tasks again when faced with highly variable measures for that particular task. Therefore, we assume – in the context of neuroadaptive Bayesian optimization – the different levels of within-task variance between groups is only practically meaningful when having a large effect size.

Given the non-significant group-level LME B.5 result, we tested for equivalence between the groups regarding motion differences (as measured by the mean framewise displacement (FD) per run) by conducting TOST against equivalence bounds of $\Delta L = -0.2$ mm to $\Delta U = 0.2$ mm on a raw scale. These bounds were chosen based on the well-established threshold of FD > 0.2 mm for scrubbing time points in resting-state fMRI data (Power *et al.*, 2012, 2014). While this threshold is used for flagging individual data points in resting-state fMRI data, the FD threshold is commonly much higher for task fMRI (Siegel *et al.*, 2013) as task-based activation analyses are less severely affected by motion than resting-state functional connectivity analyses. However, we opted for the stricter threshold, as the equivalence test is performed on the group-level differences in mean FD values per run and not individual data points. Importantly, it should be noted that the effects of motion on task-based activations were minimized in the real-time analysis of fMRI data as we performed rigorous motion scrubbing and correction on the extracted network time courses as detailed in Supplementary Methods 3.

Given the non-significant group-level LME B.2 results, we tested for equivalence between the groups regarding difference in task-induced (de)activation in the FPN and DMN by conducting TOST against equivalence bounds of $\Delta L = -0.5$ to $\Delta U = 0.5$ in standardized effect sizes (Cohen's d) for both networks. These bounds we chosen based on the literature: differences in intra-network functional connectivity in resting-state fMRI between stroke patients (n=25) and age-matched healthy controls (n=22) were reported to be high for regions in the FPN (d=-0.79/-1.09/-1.25) and moderate for regions in the DMN (d=-0.79/0.69) (Wang *et al.*, 2014). Given that we are interested group-level differences in task-induced FPN and DMN (de)activation, we could hypothesize the effect sizes to be larger than those reported in resting-state fMRI. However, we chose a conservative approach given the low sample size in (Wang *et al.*, 2014) and settled on moderate effect size bounds of 0.5.

Supplementary Results 1: Supporting analyses of language and motor network

To test this assumption, we repeated the above-mentioned analyses for a left-lateralized language network (i.e., Component 05 from (Yeo et al., 2014)). As hypothesised, we found the same results: patients' language network profiles are more dissimilar to each other than are controls to each other (t = -6.04, p = .017). Patients are also more dissimilar among each other than when comparing them with controls' profiles (t = -2.53, p = .024). By contrast, when focusing our analysis on a specific functional network associated with motor function (i.e., Component 01 from (Yeo et al., 2014)), we do not find that patients' profiles compared to each other are more dissimilar than controls compared to each other (t = 0.1, p=.568), or that patients are more dissimilar to each other than when compared to controls (t = -0.45, p = .382).

Supplementary References

Greve DN, Fischl B. Accurate and robust brain image alignment using boundary-based registration. NeuroImage 2009; 48: 63–72.

Jefferies E, Patterson K, Jones RW, Lambon Ralph MA. Comprehension of concrete and abstract words in semantic dementia. Neuropsychology 2009; 23: 492–9.

Jenkinson M, Bannister P, Brady M, Smith S. Improved optimization for the robust and accurate linear registration and motion correction of brain images. NeuroImage 2002; 17: 825–41.

Jenkinson M, Beckmann CF, Behrens TEJ, Woolrich MW, Smith SM. FSL. NeuroImage 2012; 62: 782–90.

Jenkinson M, Smith S. A global optimisation method for robust affine registration of brain images. Med Image Anal 2001; 5: 143–56.

Krishnan S, Leech R, Mercure E, Lloyd-Fox S, Dick F. Convergent and Divergent fMRI Responses in Children and Adults to Increasing Language Production Demands. Cereb Cortex 2015; 25: 3261–77.

Lakens D, Scheel AM, Isager PM. Equivalence Testing for Psychological Research: A Tutorial: [Internet]. Adv Methods Pract Psychol Sci 2018[cited 2020 Oct 12] Available from: https://journals.sagepub.com/doi/10.1177/2515245918770963

Lorenz R, Violante IR, Monti RP, Montana G, Hampshire A, Leech R. Dissociating frontoparietal brain networks with neuroadaptive Bayesian optimization. Nat Commun 2018; 9: 1227.

Luccia GD, Ortiz KZ. Association between Aphasia and Acalculia: Analytical Cross-Sectional Study. Int J Clin Med 2016; 7: 1–9.

McKenna P, Warrington E. Graded naming test. NFER-Nelson; 1983

Phillips M, Rogers P, Haworth J, Bayer A, Tales A. Intra-Individual Reaction Time Variability in Mild Cognitive Impairment and Alzheimer's Disease: Gender, Processing Load and Speed Factors [Internet]. PLoS ONE 2013; 8[cited 2020 Oct 13] Available from: https://www.ncbi.nlm.nih.gov/pmc/articles/PMC3677873/

Power JD, Barnes KA, Snyder AZ, Schlaggar BL, Petersen SE. Spurious but systematic correlations in functional connectivity MRI networks arise from subject motion. Neuroimage 2012; 59: 2142–54.

Power JD, Mitra A, Laumann TO, Snyder AZ, Schlaggar BL, Petersen SE. Methods to detect, characterize, and remove motion artifact in resting state fMRI. NeuroImage 2014; 84: 320–41.

Rasmussen CE, Williams CKI. Gaussian Processes for Machine Learning. Cambridge, Mass: MIT Press; 2006

Siegel JS, Power JD, Dubis JW, Vogel AC, Church JA, Schlaggar BL, et al. Statistical improvements in functional magnetic resonance imaging analyses produced by censoring high-motion data points. Hum Brain Mapp 2013; 35: 1981–96.

Sliwinska MW, Violante IR, Wise RJS, Leech R, Devlin JT, Geranmayeh F, et al. Stimulating Multiple-Demand Cortex Enhances Vocabulary Learning. J Neurosci 2017; 37: 7606–18.

Smith SM. Fast robust automated brain extraction. Hum Brain Mapp 2002; 17: 143–55.

Srinivas N, Krause A, Seeger M, Kakade SM. Gaussian Process Optimization in the Bandit Setting: No Regret and Experimental Design. In: Proceedings of the 27th International Conference on Machine Learning. 2010. p. 1015–22

Wang C, Qin W, Zhang J, Tian T, Li Y, Meng L, et al. Altered Functional Organization within and between Resting-State Networks in Chronic Subcortical Infarction: [Internet]. J Cereb Blood Flow Metab 2014[cited 2020 Oct 14] Available from: https://journals.sagepub.com/doi/10.1038/jcbfm.2013.238?url_ver=Z39.88-2003&rfr_id=ori%3Arid%3Acrossref.org&rfr_dat=cr_pub++0pubmed

Yeo BTT, Krienen FM, Eickhoff SB, Yaakub SN, Fox PT, Buckner RL, et al. Functional Specialization and Flexibility in Human Association Cortex. Cereb Cortex 2014: bhu217.
